# Fabrication and Model Characterization of the Electrical Conductivity of PVA/PPy/rGO Nanocomposite

**DOI:** 10.3390/molecules27123696

**Published:** 2022-06-08

**Authors:** Oladipo Folorunso, Moses Oluwafemi Onibonoje, Yskandar Hamam, Rotimi Sadiku, Suprakas Sinha Ray

**Affiliations:** 1 Department of Electrical Engineering, French South African Institute of Technology (F’SATI), Tshwane University of Technology, Pretoria 0001, South Africa; hamama@tut.ac.za; 2 Department of Electrical, Electronic and Computer Engineering, Afe Babalola University, Km 8.5, Afe Babalola Way, Ado-Ekiti 260213, Nigeria; onibonojemo@abuad.edu.ng; 3 École Supérieure d’Ingénieursen Électrotechnique et Électronique, Cité Descartes, 2 Boulevard Blaise Pascal, 93160 Noisy-le-Grand, France; 4 Department of Chemical, Institute of Nano Engineering Research (INER), Metallurgy and Material Engineering, Tshwane University of Technology, Pretoria 0001, South Africa; sadikur@tut.ac.za; 5 Centre for Nanostructures and Advanced Materials, DSI-CSIR Nanotechnology Innovation Centre, Council for Scientific and Industrial Research, Pretoria 0001, South Africa; rsuprakas@csir.co.za; 6 Department of Chemical Sciences, University of Johannesburg, Doornfontein, Johannesburg 2028, South Africa

**Keywords:** polyvinyl alcohol, polypyrrole, graphene, electrical conductivity, energy storage, models, percolation threshold

## Abstract

Owing to the numerous advantages of graphene-based polymer nanocomposite, this study is focused on the fabrication of the hybrid of polyvinyl alcohol (PVA), polypyrrole (PPy), and reduced graphene-oxide. The study primarily carried out the experimentation and the mathematical analysis of the electrical conductivity of PVA/PPy/rGO nanocomposite. The preparation method involves solvent/drying blending method. Scanning electron microscopy was used to observe the morphology of the nanocomposite. The electrical conductivity of the fabricated PVA/PPy/rGO nanocomposite was investigated by varying the content of PPy/rGO on PVA. From the result obtained, it was observed that at about 0.4 (wt%) of the filler content, the nanocomposite experienced continuous conduction. In addition, Ondracek, Dalmas s-shape, dose–response, and Gaussian fitting models were engaged for the analysis of the electrical transport property of the nanocomposite. The models were validated by comparing their predictions with the experimental measurements. The results obtained showed consistency with the experimental data. Moreover, this study confirmed that the electrical conductivity of polymer-composite largely depends on the weight fraction of fillers. By considering the flexibility, simplicity, and versatility of the studied models, this study suggests their deployment for the optimal characterization/simulation tools for the prediction of the electrical conductivity of polymer-composites.

## 1. Introduction

The ability to control the properties of polymers by the inclusion of nano-fillers gives rise to the fabrication of devices for energy storage, medical applications, computer systems, civil construction, automobile parts, and industrial equipment. For instance, Al-Zu’bi et al. [1] stated that in the process of retrofitting, high-performing polymer-composite can be achieved when carbonaceous fillers are composited with polymers and cement. The excellent electrochemical activity, electrical conductivity, large surface area, and ion transition path are some of the classified properties of conducting polymer for energy storage [2]. The properties of polythiophene doped Iron (III) chloride and polyamide 6 (PA6) make the composite suitable for the manufacturing of solar cells [3]. The composites of polylactic acid, polydopamine, and polyurethane with some compatible inorganic materials are often used for antibacterial infections [4]. The electric double-layer capacitor performances of PVA-doped-potassium-iodide- and -glycerol, has been reported by Aziz et al. [5]. As a biocompatible polymer, PVA is environmentally benign with relatively low cost, and it is suitable for the manufacturing of electrodes for supercapacitors and batteries [6,7,8,9].

The invaluable need for energy storage in the present and future generation due to the dilemma caused by the use of fossil fuel fosters the urgency for the development of a plethora of types of electrodes for electrochemical energy storage. Amongst others, polymer-composites for supercapacitors and batteries are envisaged to ravage the problems confronting the present-day electrodes. Conducting polymers such as polypyrrole and polyaniline possess high electrical conductivity and good redox reaction, but poor cyclability sets them at bay. However, the hybrid or composite of conducting polymers with two-dimensional materials, such as graphene and its derivative, produces materials having excellent electrochemical, thermal, and mechanical properties for energy storage electrodes [10,11,12]. Graphene is electrically conductive, as well as chemically, thermally, and mechanically stable.

The electrical conductivity of polymer-composites largely depends on the preparation methods and the intrinsic conductivity of the individual materials [13,14]. Of further concern is the percolation trend of polymer-composites’ electrical conductivity. The description of the electrical conductivity of polymer-composites cannot be singly achieved by experimentation technique, hence the need for analytical and descriptive models. While envisaging polymer-composites for energy storage, models must be meaningfully engaged in order to produce optimized and less costly electrodes. In our previous study, the electrical conductivity models for different polymer-composites were discussed [15]. The network usually formed by the mixture of filler and matrix can be easily analyzed and characterized by some set of well-formulated mathematical equations. In a study by Clingerman [16], the performances of statistical percolation, thermodynamic, structure-oriented, and geometry percolation models for the prediction of the electrical conductivity of polymer-composite were reported. The Mamunya model [17], which is an example of a thermodynamic model, was described by Clingerman as most suitable for the prediction of the electrical conductivity of polyacrylonitrile filled carbon fibers. Several authors [18,19,20,21] have enunciated the dependency of polymer-composites’ conductivity on some parameters. Of course, previous models are viable and useful for the prediction of the electrical conductivity of polymer-composite. However, further study on most suitable models cannot be overstated due to the many parameters on which the properties of polymer-composites depend.

The purpose of this study is to experimentally investigate the electrical property of the nanocomposite of polyvinyl alcohol (PVA), polypyrrole (PPy), and reduced graphene oxide (rGO)—PVA/PPy/rGO nanocomposite. Secondly, the characterization of the electrical conductivity of PVA/PPy/rGO nanocomposite by using various models as discussed in Section 1.1, Section 1.2, Section 1.3 and Section 1.4 was conducted. The models were validated by using the experimental measurements, and their results were presented accordingly. The results showed that the models are versatile for the prediction of the electrical conductivity of polymer-composites. However, further modification may be required in order to achieve high accuracy in subsequent studies.

### 1.1. Ondracek Model

The model developed by Ondracek [22], considered four (4) parameters, which are: shape factor, orientation, volume fraction, and conductivity. The model is given by Equation (1).

(1) $$1-C_{D}=\frac{\phi_{D}-\phi_{c}}{\phi_{D}-\phi_{m}}{\left(\frac{\phi_{m}}{\phi_{c}}\right)}^{h}{\left(\frac{\phi_{c}+g\phi_{D}}{\phi_{m}+g\phi_{D}}\right)}^{k}$$

where $h,\;k,\;g=f\left(F_{D},\cos^{2}\alpha_{D}\right)$, $C_{D}\;\left(0\leq C_{D}\leq 1\right)$ is the volume fraction of filler, $\phi_{D}$ and $\phi_{m}$ are the filler and matrix conductivity phase, $F_{D\;}\left(0\leq F_{D}\leq 0.5\right)$ is the filler shape factor, and $\cos^{2}\alpha_{D}\;\left(0\leq \cos^{2}\alpha_{D}\leq 1\right)$ is the orientation factor. The Ondracek model is not often considered an effective and appropriate model for the prediction of polymer-composites’ electrical conductivity due to its inability to predict composite electrical conductivity when the filler phase conductivity is of higher magnitude than the matrix counterpart. However, the model can be linearly re-parameterized to give a better prediction accuracy. By expanding Equation (1) while ignoring the effects of exponential parameters h and k, Equation (2) is as given.

(2) $$\frac{g\phi_{D}^{2}}{\phi_{m}}-\phi_{m}-C_{D}\phi_{D}-\frac{gC_{D}\phi_{D}^{2}}{\phi_{m}}+C_{D}\phi_{m}+gC_{D}\phi_{D}=\frac{g\phi_{D}^{2}}{\phi_{c}}-\phi_{c}$$

Assuming: $g\phi_{D}^{2}\ll \;\phi_{c}^{2}$, Equation (3), is as presented:

(3) $$\phi_{c}=C_{D}\phi_{D}\left(1-g\right)+\phi_{m}\left(1-C_{D}\right)-\frac{g\phi_{D}^{2}}{\phi_{m}}\left(1-C_{D}\right)$$

Equation (3) is equivalent to Equation (4).

(4) $$\sigma =v_{f}\sigma_{f}\left(1-g\right)+\sigma_{p}\left(1-v_{f}\right)-\frac{g\sigma_{f}^{2}}{\sigma_{p}}\left(1-v_{f}\right)$$

where $v_{f}$, $\sigma_{f}$, $\sigma_{p}$, and σ, are the filler volume fraction, filler, polymer, and composite conductivities; and $g=1-\frac{1}{2F_{D}+\left(1-3F_{D}\right)\cos^{2}\alpha_{f}}$. Equation (4) clearly shows the importance of filler volume fraction in the determination of the electrical conductivity of the nanocomposite. That is, the composite conductivity equals to the conductivity of filler when the volume fraction of the filler equals zero. The linearized form of Equation (1) is given by Equation (4). On a logarithm scale, Equation (4) can be used to measure and predict the electrical conductivity of polymer-composites to a certain degree of accuracy.

### 1.2. Dalmas s-Shape Model

In its original form, the s-shape function proposed by Dalmas [23], for polymer-composites electrical conductivity, is as given in Equation (5).

(5) $$S\left(\psi \right)=\sigma_{s}+\frac{\sigma_{cE}-\sigma_{s}}{1+e^{-a\psi +b}}$$

where $\sigma_{cE}$, $\sigma_{s}$, $S\left(\psi \right)$, are the contact, surfactant, composite conductivities; ψ, a, and b are the volume fraction and fitting parameters. Dalmas provided the performances of Equation (5) by using it to describe the experimental data of MWNT-polymer nanocomposites. Equation (6) is equivalent to Equation (5).

(6) $$\sigma =\sigma_{1}+\frac{\sigma_{2}}{1+e^{-av_{f}+b}}$$

where $\sigma_{1}$ and $\sigma_{2}$ are the initial and maximum electrical conductivities of the polymer-composites.

### 1.3. Dose–Response Model

A dose–response model is a sigmoidal model that has been extensively used in pharmaceutical and clinical industries to measure the cause and effect of drug, illness, death, and diseases [24]. For instance, Park et al. [25] studied the treatment of tumor via radiotherapy exposure by using dose–response theory. In the study, Park et al. discovered that a high dose of radiation gives a better treatment response for hepato-cellular carcinoma. Furthermore, Li et al. [26], carried out a dose–response investigation on fat distribution and incidental liver cancer. The result of the investigation showed that liver disease increases the chance of atypical obesity. In mathematical terms, dose is an independent parameter (cause), while response is the dependent parameter (effect). The dose–response theory can be used to determine the properties of polymer-composites by assuming that the various determining factors of the properties are associated to dose parameters, while the response is the resulting property. Rahaman et al. [27] previously studied the applicability of sigmoidal equations to polymer-composites’ electrical conductivity determination.

According to Ritz et al. [28], the four-parameter-log-logistic model, presented in this study as Equation (7), is the most frequently used dose–response equations.

(7) $$\sigma \left(v_{f},\left(\sigma_{1},\sigma_{2},\;c,d\right)\right)=\sigma_{1}+\frac{\sigma_{2}-\sigma_{1}}{1+e^{\left(c\left(\log\left(v_{f}\right)-\log\left(d\right)\right)\right)}}$$

Herein, c is the slope or steep factor and d is the effective dose parameter. From the experimental data presented in this study, the performance of the dose–response model of Equation (7) was slightly modified for better accuracy. The modified form of Equation (7) is as presented in Equation (8). Another possible way to achieve a better accuracy from the prediction of Equation (7) is to critically set the initial values of the dependent parameters from the simulation code.

(8) $$\sigma \left(v_{f},\left(\sigma_{1},\sigma_{2},\;c,d\right)\right)=\sigma_{1}+\frac{\sigma_{2}-\sigma_{1}}{1+e^{-clog\left(v_{f}\right)}e^{-clog\left(d\right)}}$$

### 1.4. Gaussian Fitting Model

The Gaussian model is another important predictive model considered in this study. The versatility of the Gaussian model has been proved by many researchers [29,30,31,32]. It is, therefore, important to test how useful Gaussian fitting model can be in predicting the property of polymer-composite, viz.: electrical conductivity. Amongst others, filler orientation, synthesis method, initial conductivity of matrix/filler, weight fraction, and shape are factors that contribute to the electrical property of polymer nanocomposites. The Gaussian equation presented by Equation (9), is a three-parameter equation.

(9) $$\sigma =\overset{N}{\underset{n}{\sum }}\sigma_{n}e^{-{\left(\frac{x-k_{n}}{z_{n}}\right)}^{2}}$$

where $z_{n}$ and $k_{n}$ are factors relating to the percolation threshold and saturating weight fraction; $\sigma_{n}$ is the electrical conductivity of filler/matrix.

## 2. Results

**Figure 1:**
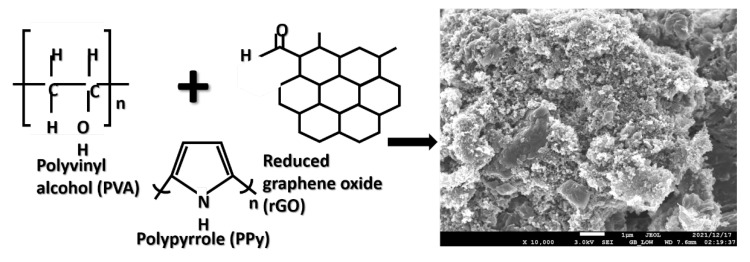
The structures and the morphology of PVA/PPy/rGO nanocomposite (0.4 (wt%)).

A typical micrograph of PVA/PPy/rGO nanocomposite is shown in Figure 1. The micrograph of polymer-composite is very important in order to observe the interaction between the fillers and matrices. As shown in Figure 1, the ternary materials are homogenously mixed; however, the interaction was by van der Waals and π−π interactions [33].

In the preparation and electrical conductivity measurement of polymer-composites, the most important parameter of interest is the percolation threshold. A polymer-composite with a high percolation threshold suggests a low electrically conductive composite; such a composite will require a high-volume fraction of filler, which is synonymous with a high cost of production. On the other hand, a low percolation threshold gives rise to an increase in the electrical conductivity, reduces filler weight and improves mechanical and chemical properties of the polymer-composites. One of the determinants of polymer-composites’ percolation threshold is the preparation method. The interface between filler/filler and filler/matrix, are functions of the morphology of the composite, a factor which can be controlled by the hybridization and synthesis methods.

**Figure 2:**
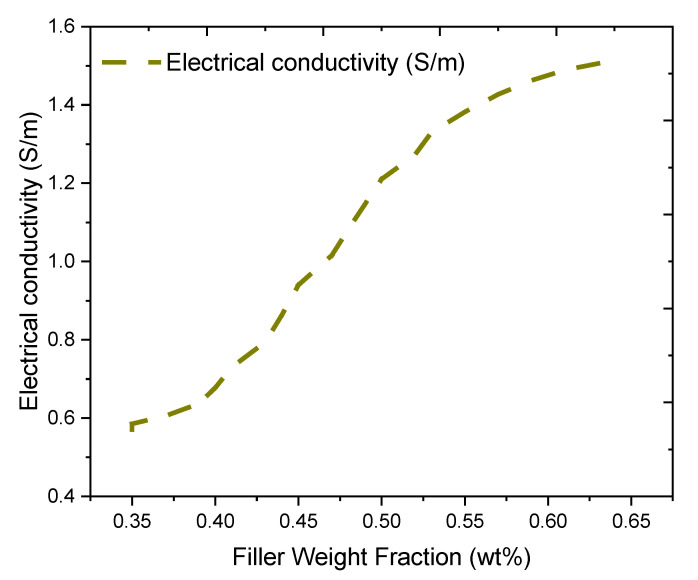
Electrical conductivity measurement of PVA/PPy/rGO nanocomposites.

The electrical conductivity measurement of the fabricated PVA/PPy/rGO nanocomposite is as shown in Figure 2. As it can be seen from the figure, the electrical conductivity of the nanocomposite gained a continuous conductive path at about 0.4 (wt%) of the hybrid filler. With respect to the recorded low percolation threshold, it can be ascertained that the electrical property of the materials has been improved. The low percolation threshold also suggests that the filler possesses a high aspect ratio [34].

### Experimental Data and Modeling Analysis

The models previously mentioned in Section 1.1, Section 1.2, Section 1.3 and Section 1.4, were used to describe the electrical conductivity data of the fabricated PVA/PPy/rGO nanocomposite. The description of the experimental data is important in order to theoretically formulate and propose models for the prediction of the electrical conductivity of polymer-composites. It is also necessary to mention that the models can save the time and cost of preparing polymer-composite.

**Figure 3:**
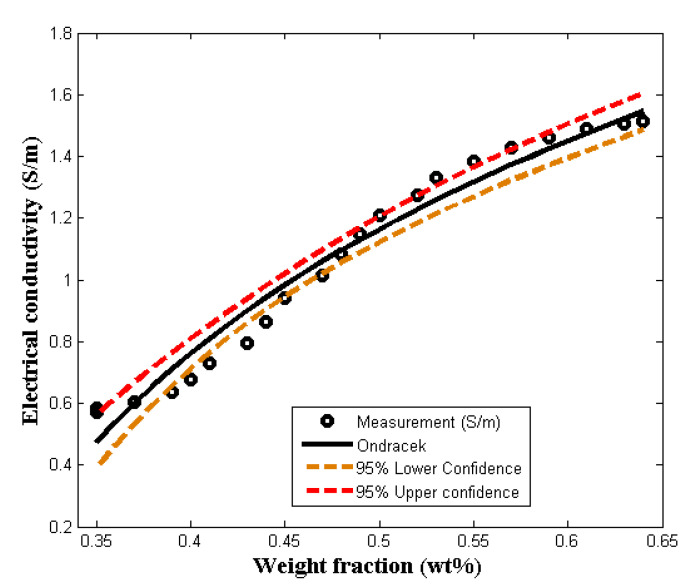
Comparing the linearized Ondracek model (LOM) with experimental measurement.

Figure 3 shows the comparison and the description of PVA/PPy/rGO nanocomposite conductivity data by the linearized Ondracek model. The Ondracek model is concerned with orientation of filler, shape, weight fraction, and conductivities of the filler and matrix. From the linearized Ondracek equation, the effects of the aforementioned factors are presented by $\chi_{1}$ and $\chi_{2}$, as shown in Table 1. The re-parameterized equation of Equation (4) is as shown by Equation (10); where x is the volume fraction. From the results, it can be observed that the effect of *g*-factor in the equation, which represents the shape and orientation factor, can be positive or negative. Subsequent to the fact that the model is linear, it is difficult to truly observe how the *g*-factor affects the percolation threshold of the nanocomposite. A high percolation threshold is usually associated with anisotropic orientation of fillers; otherwise, a low percolation shows that the orientation is isotropic [35]. In addition, further observation from the figure is that the model can predict the conducting region of the composite before saturation. Table 1 displays the accuracy of the model. The deviation of the model from the experimentation data is 0.03 (0.038 adjusted). The per unit errors for the calculated parameters concisely give the performance of the model. Therefore, the model is proposed for re-modification in order to give better accuracy by producing agreeable simulation data with experimental data.

(10) $$\sigma =\log\left(\chi_{1}x+\chi_{2}\left(1-x\right)\right)$$

**Figure 4:**
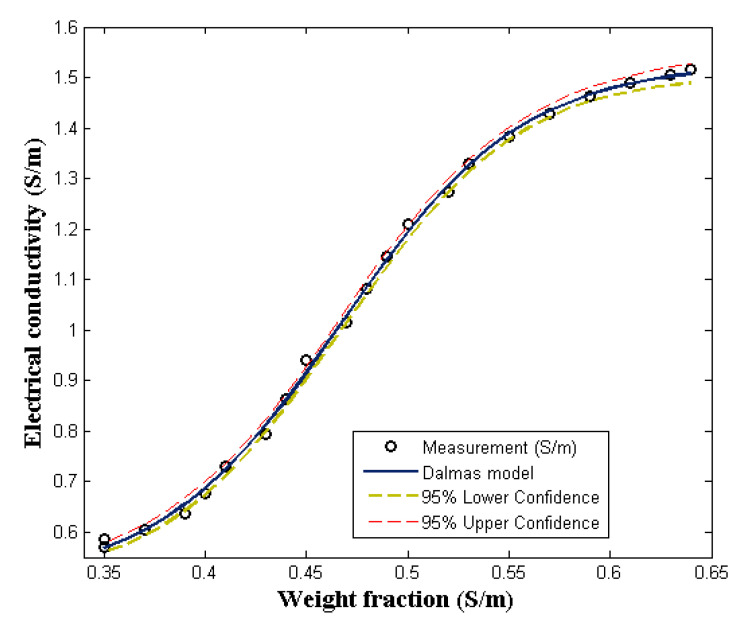
Comparing the Dalmas s-shape model (DsM) with experimental measurement.

The electrical conductivity of polymer-composite usually depicts an *s*-like shape, which can be segmented into before and after percolation threshold, and saturation regions. These regions often form *s*-shapes like graphs for most polymer-composite electrical conductivity. As shown in Figure 4, the comparison of PVA/PPy/rGO nanocomposite electrical conductivity data with the Dalmas s-shape model, is elucidated. In Equation (6), the Dalmas s-shape equation having $\sigma_{1}$ as the matrix conductivity and $\sigma_{2}$ as the saturation conductivity of the composite is presented. According to the literature, the fitting parameters, a and b, account for the effects of aspect ratio and shape factor. The aspect ratio and shape factor determine the point at which the polymer-composite changes from linear conductivity to the point of saturation [36]. As shown in Figure 4, the Dalmas s-shape model fits the experimental data well, and it was able to trace all the conductivity regions of the composite. The accuracy of the prediction can be found in Table 2. From the results presented in Table 2, it can be asserted that the model is in good agreement with the experimental data. Provided by Equation (11) is the Dalmas *s*-shape equivalent electrical conductivity predictive model for PVA/PPy/rGO nanocomposite. This model can be generalized to predict or determine the electrical conductivity of polymer-composites other than PVA/PPy/rGO nanocomposite. The model is simple, unique, and versatile, and it can be deployed into computer system application for the prediction of the electrical conductivity of polymer-composites.

(11) $$\sigma =0.5+\frac{1.03}{1-e^{-22.37x+10.46}}$$

**Figure 5:**
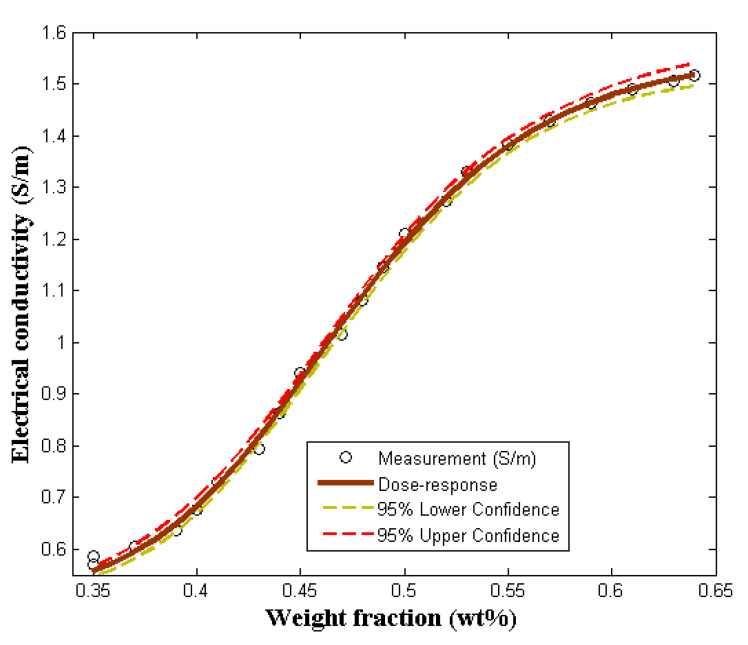
Comparing the dose–response model with experimental measurement.

Figure 5 compares the dose–response model with the experimental data. The initial/maximum conductivity, orientation factor, and aspect ratio are the parameters considered in the model. In the determination of the electrical conductivity of polymer-composite, the effect of the aspect ratio and orientation is inverse proportionality to percolation threshold. As it is shown in Figure 5, the calculated value for the percolation threshold is the same with the experimental value. The model agrees with the experimental data. This is evidence that a dose–response model can be effectively applied to predict and characterize the electrical behavior of polymer-composite [27]. In addition, Table 3 provides the performance and accuracy of the dose–response model considered in this study. In order to further simplify the model for the prediction of the electrical conductivity of PVA/PPy/rGO nanocomposite, Equation (12) is as presented. The correlation between the experimental data and the dose–response model as applied to the present study is an indication that the model can be generalized for the prediction of polymer-composite electrical conductivity of any type.

(12) $$\sigma =0.5+\frac{1.07}{1+7.40e^{9.79\log x}}$$

**Figure 6:**
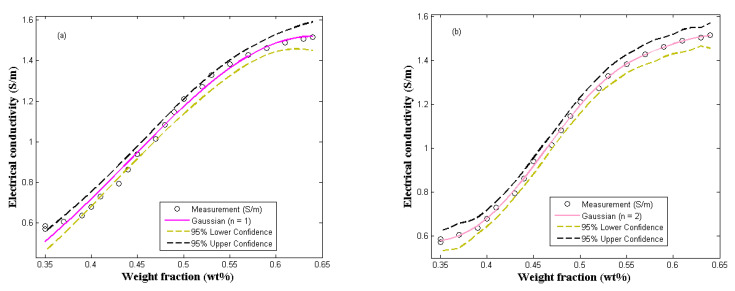
Comparing the Gaussian model with experimental measurement (**a**) n = 1 (**b**) n = 2.

Moreover, according to the experimental and the Gaussian model results shown in Figure 6, respectively, the electrical conductivity of polymer-composites depends largely on the quantity of fillers in the matrix [34]. The Gaussian model is a fascinating and simple model, which is suitable for the prediction and classification of random data. At micro-scale, polymer-composites are heterogeneous; therefore, their data can be classified as random data [37]. From the results shown in Figure 6, it can be seen that the Gaussian model is appropriate for the prediction of the electrical conductivity of polymer-composites. However, for n=1 (Figure 6a), the model was moderately adequate to predict the percolation threshold of PVA/PPy/rGO nanocomposite. Figure 6b clearly showed that at n=2, the model accurately predicts the nanocomposite conductivity. Moreover, Table 4 and Table 5 give the performance and accuracy of the model. Equations (12) and (13) are the Gaussian predictive models developed for the study experimental data.

(13) $$\sigma =1.52e^{-{\left(\frac{x-0.64}{0.28}\right)}^{2}}\;n=1$$

(14) $$\sigma =0.138e^{-{\left(\frac{x-0.346}{0.032}\right)}^{2}}+1.505e^{-{\left(\frac{x-0.62}{-0.244}\right)}^{2}}\;n=2$$

## 3. Materials and Methods

The graphene (rGO) used was obtained from the CeNam-CSIR, Pretoria, South Africa. The polypyrrole with CAS No: 30604-81-0; solid, 20 wt% carbon content loading and >300 °C melting point, was purchased from Sigma Aldrich, Gauteng, South Africa. Polyvinyl alcohol with CAS No: 9002-89-5 was purchased from Sigma Aldrich, South Africa. A $\bar{M_{w}}$ of between 146,000–186,000, 99+% hydrolyzed, hydrophilic, and crystalline power, are the properties of the polyvinyl alcohol. Moreover, acetone was purchased from Sigma Aldrich and deionized water was sourced from the CSIR, Pretoria laboratory. Refer to [19] for the electrical conductivity measurement method.

### Fabrication Method

The fabrication of PVA/PPy/rGO nanocomposite was achieved by solvent blending/drying method. The first sample was produced by the following procedures. A total of 300 mg of PVA was measured into a beaker containing 20 mL of deionize water, and it was magnetically stirred for 2 h. Secondly, a 100 mg of rGO was dispersed in 300 mL of acetone/deionized water and ultra-sonicated for 1/3 h. In addition, 100 mg of PPy was added to the rGO solution and further ultra-sonicated for 1/3 hr. The ultra-sonicated mixture of PPy/rGO was loaded onto the dissolved PVA (300 mg) and rigorously stirred for more than 12 h. The resultant solution of the nanocomposite was washed by using acetone and deionize water. Moreover, the nanocomposite was vacuum-dried at 60 °C for 12 h. Moreover, the preparation of the other samples followed the same process; the PVA content was kept constant, while PPy and rGO contents were varied.

## 4. Conclusions

Herein, the electrical conductivity of PVA/PPy/rGO nanocomposite was experimentally and mathematically studied. The concept adopted in this research can usher in new insights into the development of highly efficient energy storage electrodes. The mathematical equations employed for the conductivity characterization of PVA/PPy/rGO nanocomposite showed results that correlate with the experimental measurements. The Gaussian, Dalmas s-shape, and dose–response models showed results, which are closer to the experimental measurement. However, the limitation of the models is that initialization of values for the dependent parameters is very difficult. Further studies would endeavor to provide more insights into how these models can be effectively used to characterize the electrical properties of polymer-composites, more accurately.

## Figures and Tables

**Table 1:** Linearized Ondracek model performance data.

Model	Parameters	Parameter Values	Standard Error	Per-Unit Standard Error	R^2^	R^2^-adj
Ondracek	$\chi_{1}$	8.51	0.27	0.03	0.967	0.962
	$\chi_{2}$	−2.11	0.21	0.09		

**Table 2:** Dalmas s-shape performance data.

Model	Parameters	Parameter Values	Standard Error	Per-Unit Standard Error	R^2^	R^2^-adj
Dalmas s-shape	$\sigma_{2}$	1.02	0.008	0.007		
	a	22.37	0.462	0.021	0.9989	0.9988
	b	10.46	0.203	0.019		

**Table 3:** Dose–response performance data.

Model	Parameters	Parameter Values	Standard Error	Per-Unit Standard Error	R^2^	R^2^-adj
Dose–response	$\sigma_{2}-\sigma_{1}$	1.07	0.01	0.01		
	c	−9.79	0.24	0.03	0.9986	0.9984
	$e^{-clog\left(d\right)}$	7.40	0.21	0.03		

**Table 4:** Gaussian fitting performance data (n = 1).

Model	Parameters	Parameter Values	Standard Error	Per-Unit Standard Error	R^2^	R^2^-adj
Gaussian	$\sigma_{1}$	1.52	0.024	0.02		
	$k_{1}$	0.64	0.011	0.02	0.9989	0.9902
	$z_{1}$	0.28	0.012	0.04		

**Table 5:** Gaussian fitting performance data (n = 2).

Model	Parameters	Parameter Values	Standard Error	Per-Unit Standard Error	R^2^	R^2^-adj
Gaussian	$\sigma_{1}$	0.138	0.033	0.236	0.9983	0.9975
	$k_{1}$	0.346	0.019	0.056		
	$z_{1}$	0.032	0.019	0.578		
	$\sigma_{2}$	1.505	0.008	0.005		
	$k_{2}$	0.620	0.004	0.007		
	$z_{2}$	−0.244	0.007	0.027		

## Data Availability

Data available from the corresponding author (O.F.).
